# Approach to Imaging of Mediastinal Masses

**DOI:** 10.3390/diagnostics13203171

**Published:** 2023-10-11

**Authors:** Jitesh Ahuja, Chad D. Strange, Rishi Agrawal, Lauren T. Erasmus, Mylene T. Truong

**Affiliations:** 1 Department of Thoracic Imaging, The University of Texas MD Andeson Cancer Center, Houston, TX 77030, USA; cdstrange@mdanderson.org (C.D.S.); ragrawal1@mdanderson.org (R.A.); mtruong@mdanderson.org (M.T.T.); 2 Department of Anatomy and Cell Biology, Faculty of Sciences, McGill University, Montreal, QC H3A 0G4, Canada; lauren.erasmus@mail.mcgill.ca

**Keywords:** mediastinum, imaging, CT, MRI

## Abstract

Mediastinal masses present a diagnostic challenge due to their diverse etiologies. Accurate localization and internal characteristics of the mass are the two most important factors to narrow the differential diagnosis or provide a specific diagnosis. The International Thymic Malignancy Interest Group (ITMIG) classification is the standard classification system used to localize mediastinal masses. Computed tomography (CT) and magnetic resonance imaging (MRI) are the two most commonly used imaging modalities for characterization of the mediastinal masses.

## 1. Introduction

**Figure 1:**
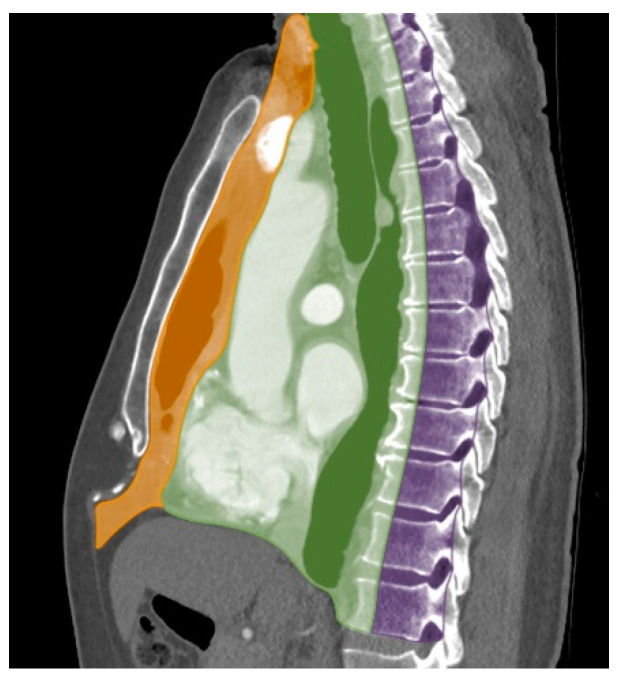
ITMIG definition of mediastinal compartments. Sagittal reformatted CT with color overlay shows mediastinal compartment borders: prevascular compartment (orange), visceral compartment (green) and paravertebral compartment (purple).

The mediastinum is a complex anatomical space between the lungs bounded superiorly by the thoracic inlet, inferiorly by the diaphragm, anteriorly by the sternum and posteriorly by the spine. The mediastinum contains vital structures that can be affected by a wide range of pathologies including neoplasm (benign and malignant), congenital and vascular abnormalities, glandular hyperplasia and lymphadenopathy. Accurate localization of the mass to a mediastinal compartment is the first and crucial step to creating a useful differential diagnosis. The mediastinal compartment classification system advocated by the International Thymic Malignancy Interest Group (ITMIG) in 2014 is the standard classification system used to localize mediastinal masses. This classification system divides mediastinum into three compartments: prevascular (anterior), visceral (middle) and paravertebral (posterior) compartments using anatomical landmarks on CT (Table 1 and Figure 1) [1,2,3,4,5].

Imaging plays an important role in the evaluation, diagnosis, staging and treatment planning of mediastinal pathologies. Mediastinal masses are commonly discovered incidentally upon chest radiography. Occasionally, mediastinal masses may be discovered upon echocardiography performed in patients with nonspecific chest pain. Echocardiography is also helpful in evaluating hemodynamic complications of intracardiac extension of mediastinal masses [6,7]. Cross-sectional imaging modalities such as computed tomography (CT) and magnetic resonance imaging (MRI) are used to evaluate the internal characteristics of the mass. It should be accurately determined if the mass is solid or cystic and assessed for the presence of fat or calcification. Post-contrast images are used to determine vascularity of the mass. This approach helps the radiologists to narrow the differential or make a specific diagnosis.

## 2. Imaging Modalities

**Figure 2:**
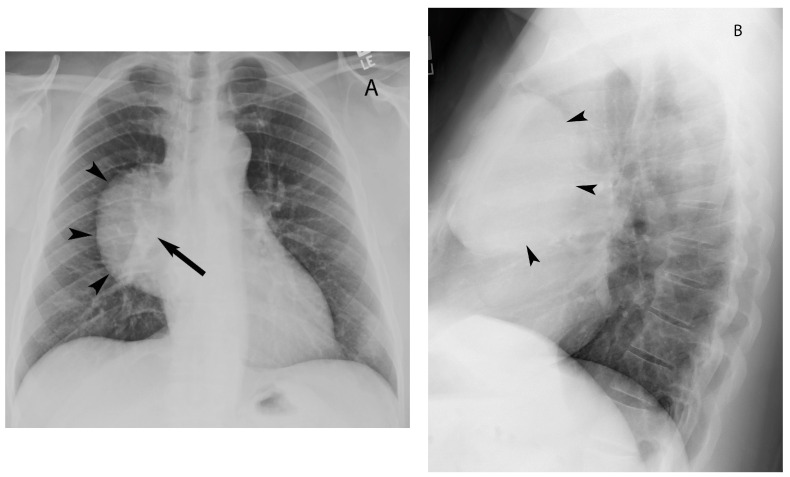
Hilum overlay sign. Chest radiograph posteroanterior view (**A**) shows well-marginated mass over right hilum (arrowheads). Hilar vessels (arrow) are seen through the mass, suggesting the mass is either anterior or posterior to the hilum. Chest radiograph lateral view (**B**) confirms the anterior location of the mass (arrowheads).

Chest radiography is often the first imaging modality to demonstrate mediastinal mass. Two view chest radiographs (PA and lateral) are particularly helpful for localizing the abnormality to a mediastinal compartment. The hilum overlay sign on frontal chest radiography helps in determining if an opacity in the region of the hilum is located within the hilum versus anterior or posterior to it. If the hilar vessels are seen through the mass, it indicates the mass does not originate in the hilum and is located either anteriorly or posteriorly to it [8] (Figure 2). The American College of Radiology Appropriateness Criteria Imaging of Mediastinal Masses recommends either CT or MRI as the next imaging study of patients with indeterminate mediastinal mass detected upon radiography [9]. CT evaluation of the mass includes location, size, attenuation (solid vs. cystic), presence of fat or calcification, relationship to adjacent structures and enhancement pattern. Additionally, CT is helpful in evaluating for lymphadenopathy, and involvement of the lungs, pleura and bone [1,2]. MRI can be used as a problem-solving tool specifically when it is difficult to distinguish cystic from solid lesions on CT and characterization of complex cystic lesions. It is also useful to differentiate thymic hyperplasia from neoplasms [10,11,12]. Fluorodeoxyglucose positron emission tomography/computed tomography (FDG-PET/CT) has a limited role in the evaluation of the mediastinal mass as it is nonspecific, and several infectious or inflammatory processes can show FDG uptake. Studies that evaluate the role of FDG-PET/CT in distinguishing low-grade thymoma, high-grade thymoma and thymic neoplasm show significant overlap when using the parameter of FDG avidity. FDG-PET/CT is routinely performed for staging and restaging of lymphoma [8,13,14].

### Prevascular (Anterior) Compartment

More than 50% of the mediastinal masses originate in the prevascular compartment [3,15,16]. The most common prevascular mediastinal mass arises in the thymus and can be divided into epithelial and nonepithelial tumors. Thymic epithelial tumors include thymomas and thymic carcinomas. Nonepithelial tumors include lymphomas, carcinoids, germ cell tumors and lipomas [2,8,17,18]. As described above, the first approach in the diagnostic algorithm is to determine if the tumor is solid or cystic. The four common solid masses in the prevascular compartment include thymomas, teratomas, thyroid tumors (goiter) and lymphomas. Based on patient age, a useful differential can be generated, as germ cell tumors are commonly seen in patients younger than 40 years of age and thymomas are commonly seen in patients older than 40 years. Purely cystic lesions in the prevascular mediastinum include thymic or pericardial cyst [15].

## 3. Cystic Lesions

**Figure 3:**
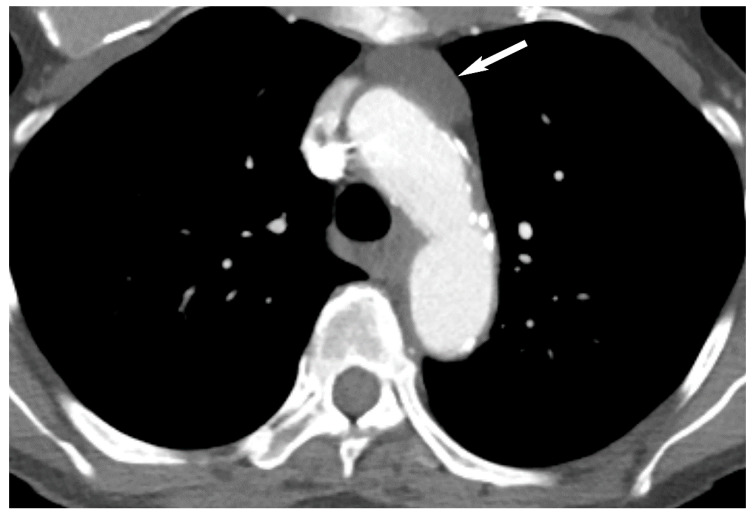
Thymic cyst. CT shows a fluid attenuating structure (15 Hounsfield units) in the prevascular mediastinum in the region of thymic bed (arrow).

**Figure 4:**
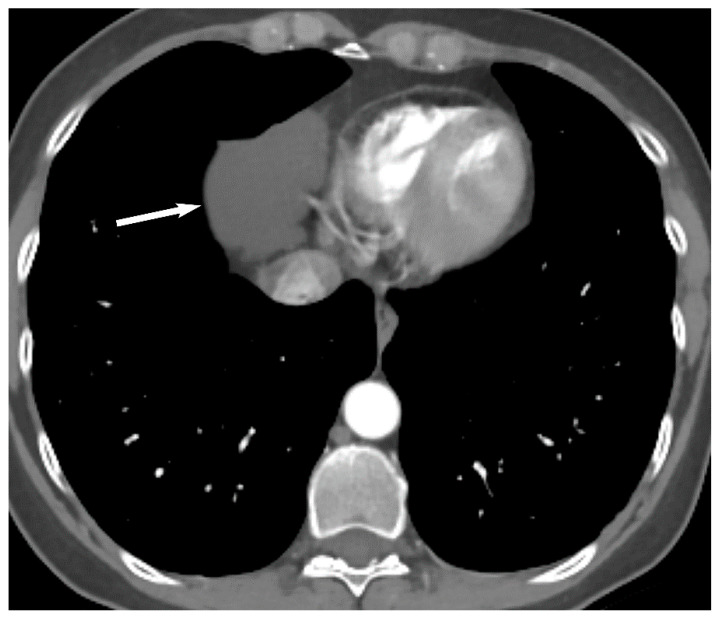
Pericardial cyst. CT shows a fluid attenuating structure (10 Hounsfield units) in the prevascular mediastinum at the right anterior cardiophrenic angle (arrow).

**Figure 5:**
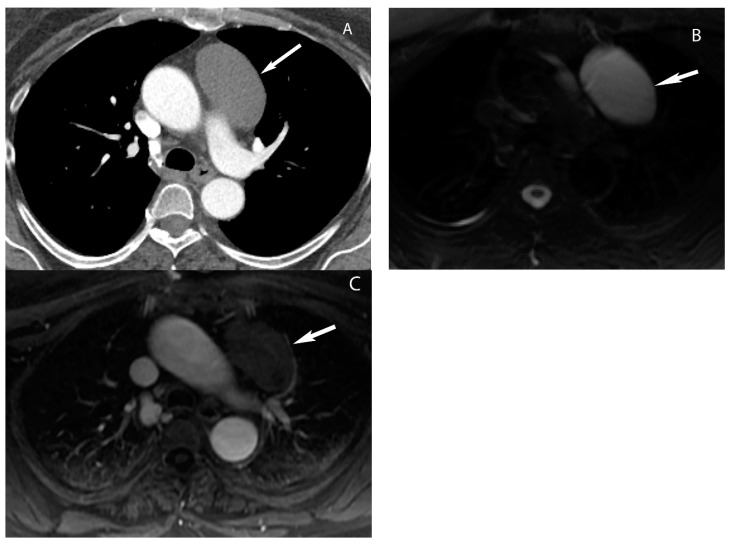
Thymic cyst. (**A**) CT shows a well-defined homogeneous structure with density slightly higher than simple fluid (40 Hounsfield units) in the prevascular mediastinum (arrow). MRI (**B**,**C**) shows T2 hyperintensity (arrow in (**B**)) and no enhancement with gadolinium (arrow in (**C**)) consistent with fluid.

**Figure 6:**
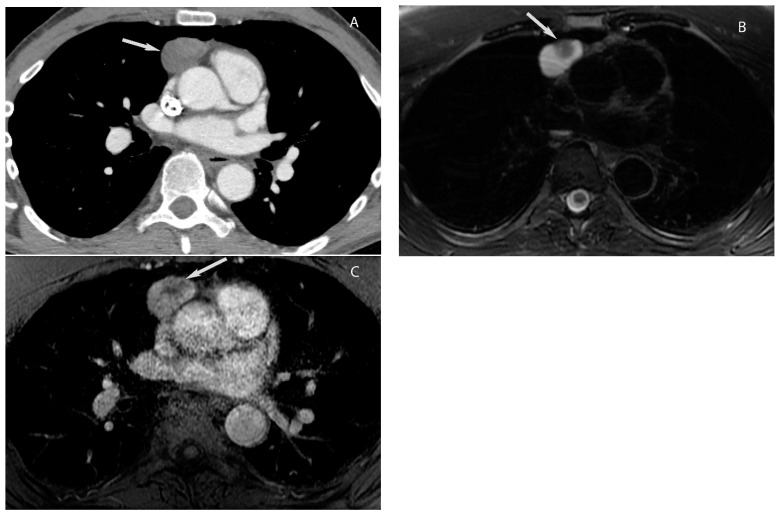
Cystic thymoma. CT (**A**) shows a mixed attenuation (cystic and solid) mass in the prevascular mediastinum. T2W MRI (**B**) shows hyperintense cystic component and hypointense solid nodule (arrow in (**B**)). Post-gadolinium MRI (**C**) shows enhancement of the solid nodule. Pathology showed cystic thymoma.

Well-defined homogeneous fluid-attenuating (0 to 20 HU) lesions in the prevascular mediastinum are of benign etiology including thymic and pericardial cysts [19,20]. These can be distinguished from one another based on their locations—thymic cysts are present in the thymic bed (Figure 3) and pericardial cysts are commonly located at the right cardiophrenic angle (Figure 4). Sometimes, thymic cysts may demonstrate attenuation values higher than simple fluid. In such cases, MRI is useful due to its ability to distinguish cystic lesions from solid lesions. They are hyperintense on T2W images and do not enhance after administration of intravenous contrast material (Figure 5) [10,11]. Multilocular cystic lesions in the prevascular mediastinum may represent multilocular thymic cyst or lymphangioma. When multilocular cystic lesion with septations or soft tissue component extends to the neck, axilla or chest wall, the possibility of lymphangioma should be considered [20,21]. The presence of soft tissue component in the cystic lesion raises the possibility of malignancy with differentials including cystic teratoma or cystic thymoma (Figure 6). The soft tissue component may enhance with intravenous contrast administration [22,23,24].

## 4. Thymic Hyperplasia

**Figure 7:**
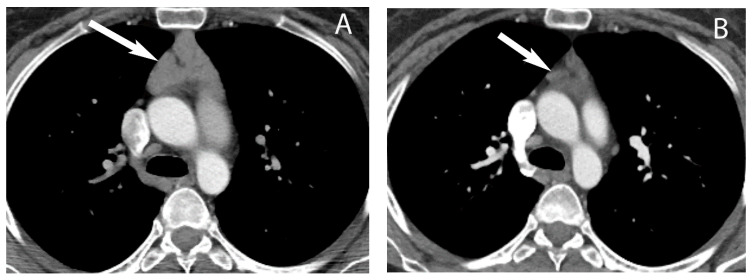
Thymic hyperplasia. CT (**A**) shows diffuse enlargement of the thymus maintaining its triangular or bilobed shape (arrow). Follow-up CT (**B**) 12 months later shows fatty involution of the thymus (arrow).

**Figure 8:**
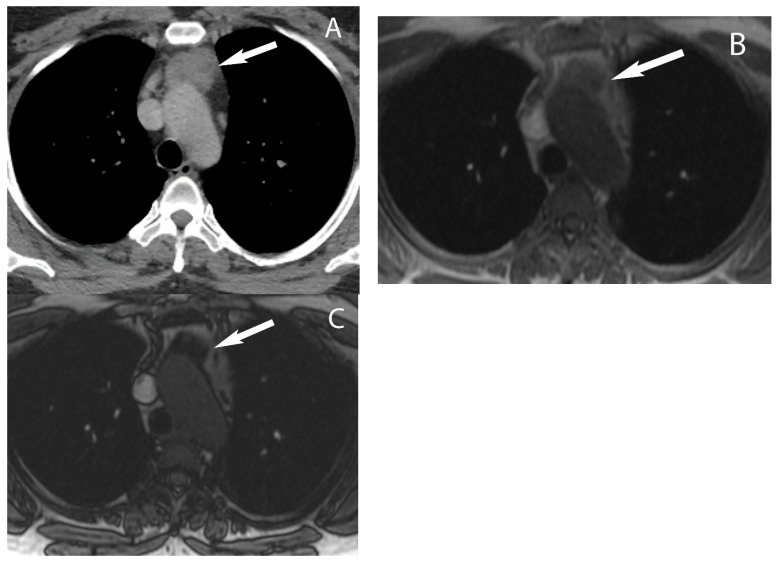
Thymic hyperplasia. CT (**A**) shows a rounded soft tissue mass in the prevascular mediastinum (arrow) in a woman with breast cancer. Differential diagnosis includes thymic epithelial neoplasm or metastatic disease. MRI chest in-phase image (**B**) shows the mass is isointense to muscle (arrow) and shows drop in signal on opposed-phase image (**C**) consistent with thymic hyperplasia.

Thymus is a lymphatic organ situated in the prevascular mediastinum. It is relatively large during infancy but gradually decreases in size and is replaced by fat as a normal aging process. In children, the thymus appears as a homogeneous smoothly marginated bilobed triangular structure of soft tissue attenuation upon CT. After puberty, attenuation gradually decreases as fatty involution takes place and usually by 40 years of age, there is near complete fatty involution of the thymus [16,25]. Rebound thymic hyperplasia can occur at any age due to physiological stress such as surgery, radiation therapy, chemotherapy, burns or steroid use. Thymic hyperplasia can manifest as diffuse symmetric enlargement of the gland maintaining its triangular bilobed shaped or a discrete mass. Thymic hyperplasia is easy to diagnose upon CT when it manifests as diffuse enlargement of the gland (Figure 7). However, when thymic hyperplasia manifests as a discrete mass, it can be a diagnostic challenge to distinguish thymic hyperplasia from thymic neoplasm upon CT. If the clinical suspicion for thymic hyperplasia is high, follow-up CT in 2–3 months can be obtained to demonstrate expected decrease in the size of the gland. Alternatively, MRI with chemical shift imaging may be helpful in distinguishing thymic hyperplasia from true thymic neoplasm. Thymic hyperplasia shows homogeneous decrease in signal intensity on opposed-phase sequence relative to in-phase sequence due to the presence of fat in contrast to thymic neoplasms that do not show any signal drop [12,18,26,27] (Figure 8).

## 5. Thymic Epithelial Neoplasms

**Figure 9:**
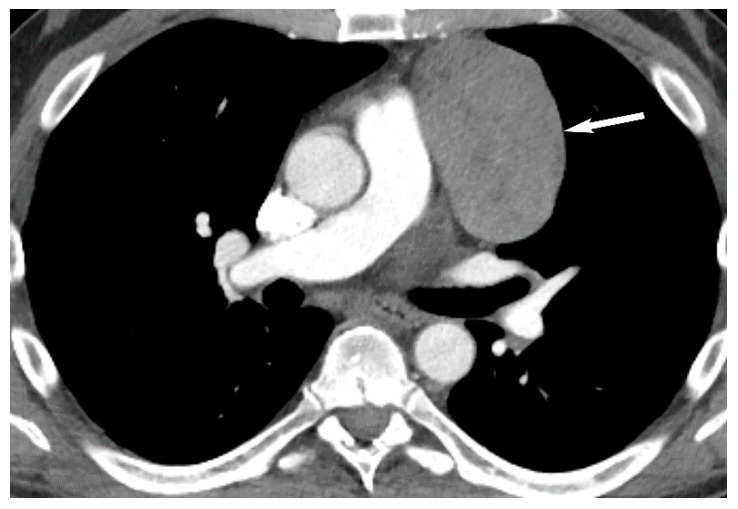
Thymoma. CT shows a well-defined homogeneous soft tissue mass in the prevascular mediastinum (arrow).

**Figure 10:**
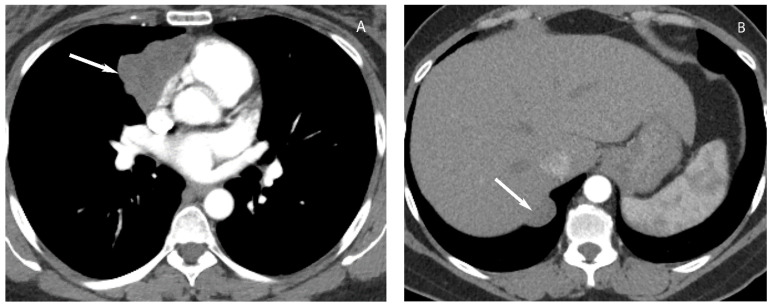
Thymoma with pleural drop metastasis. CT (**A**,**B**) shows a lobulated soft tissue mass in the prevascular mediastinum (arrow in (**A**)) and a diaphragmatic pleural nodule in the right lower chest (arrow in (**B**)).

**Figure 11:**
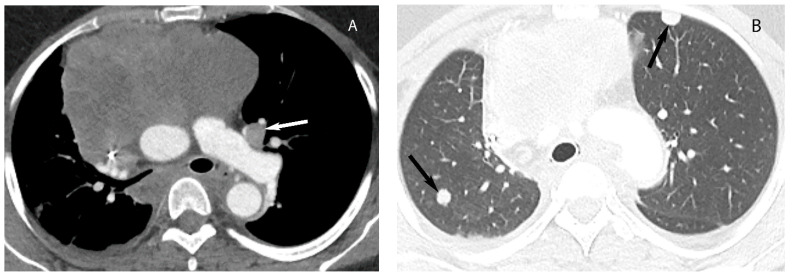
Thymic carcinoma. CT with soft tissue (**A**) and lung (**B**) window show large heterogeneous soft tissue mass in the prevascular mediastinum. Left prevascular lymphadenopathy (arrow in (**A**)) and well-marginated lung nodules (arrows in (**B**)) are compatible with metastatic disease.

Thymic epithelial neoplasms include thymoma and thymic carcinoma and are rare with an incidence of 0.2 to 1.5% of all malignancies in the United States [2,4,28]. Thymoma is the most common thymic epithelial neoplasm and the most common primary neoplasm of the prevascular mediastinum [4,12,17,18]. The peak incidence of thymoma is between 40 to 60 years of age with equal sex distribution. Presentation can be incidental on imaging in asymptomatic patients or may present with symptoms such as cough, chest pain, dysphagia or facial swelling due to mass effect or invasion of adjacent structures. Patients can also present with a variety of paraneoplastic syndromes, myasthenia gravis being the most common, and others include pure red cell aplasia, hypogammaglobulinemia and connective tissue disorders. Thymomas are usually encapsulated and slow-growing neoplasms but sometimes can invade the adjacent vascular structures, pleura and pericardium. Upon CT, noninvasive thymoma appears as a homogeneous or mildly heterogeneous soft tissue mass with well-circumscribed borders (Figure 9). When a soft tissue mass has irregular or lobular borders, or heterogeneity due to necrosis or cystic changes and intrinsic calcification, invasive thymoma should be suspected [29,30]. Intravenous contrast is useful to assess for local invasion and CT may show infiltration of the mediastinal fat, encasement or frank invasion of vascular structures, invasion of the pleura or pericardium. Pleural implants (drop metastases) may be present with invasive tumors and are highly suspicious for thymoma (Figure 10). Lymphadenopathy is typically absent in thymoma [29,30,31]. Thymic carcinomas are uncommon thymic epithelial neoplasm but are more aggressive compared to thymomas. Mean age of presentation is 50 years but in contrast to thymoma, the patients are often symptomatic at presentation. Thymic carcinomas can be indistinguishable from invasive thymomas upon imaging but should be suspected when a large heterogeneous mass with irregular margins is present in the prevascular mediastinum with invasion of adjacent structures, lymphadenopathy, pleural/pericardial effusions and distant metastasis (Figure 11) [26,29,30,32,33].

## 6. Lymphoma

**Figure 12:**
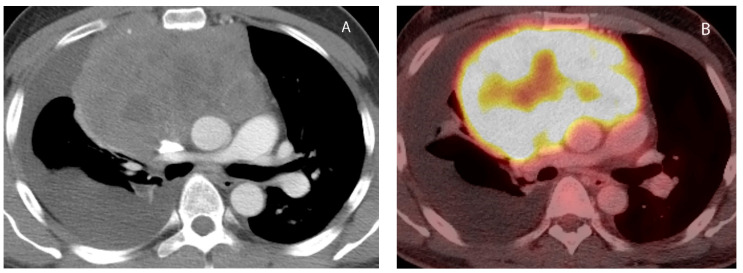
Primary mediastinal lymphoma. CT (**A**) shows large heterogeneous soft tissue mass in the prevascular mediastinum. The mass is hypermetabolic on FDG PET-CT (**B**). Biopsy showed diffuse large B cell lymphoma.

Lymphoma is the most common mediastinal tumor, accounting for about 50% to 60% of all mediastinal malignancies and can be Hodgkin or non-Hodgkin lymphoma [2,15,34]. Lymphomas arising primarily in the mediastinum are rare and comprise approximately 1% of all lymphomas [35]. Diagnosis of primary mediastinal lymphoma requires involvement of mediastinal lymph nodes, the thymus or both without involvement of any other lymph nodes or organs outside the mediastinum at the time of presentation. More commonly, lymphomas involve mediastinum secondarily as a part of systemic disease. The most common cell types with primary or secondary mediastinal lymphomas are diffuse large B-cell lymphoma and Hodgkin lymphoma. Primary mediastinal (thymic) B-cell lymphoma, gray zone lymphoma (GZL), T-cell lymphoblastic lymphoma (TCLL), mucosa associated lymphoid tissue (MALT) lymphoma and peripheral T-cell lymphoma (PTCL) are other less common cell types of lymphomas that can involve the mediastinum [35,36,37]. Upon imaging, primary mediastinal lymphoma may present as a lobulated homogeneous soft tissue mass or may show areas of low attenuation due to necrosis/cystic degeneration [8,15,38,39] (Figure 12). It might be difficult to distinguish lymphoma from other mediastinal soft tissue masses upon imaging, and biopsy is required to confirm the diagnosis. Vascular encasement or displacement without invasion is characteristic of lymphoma. When such imaging findings are seen in young patients along with B symptoms (fever, weight loss and night sweat), a diagnosis of mediastinal lymphoma should be considered. When enlarged lymph nodes are seen involving multiple mediastinal lymph node stations in the setting of extrathoracic lymphadenopathy, secondary involvement of systemic lymphoma should be considered [2,15]. F18-fluorodeoxyglucose-positron emission tomography/computed tomography (FDG-PET/CT) is the modality of choice for the initial staging and ongoing surveillance of lymphoma during treatment. It may be helpful in identifying occult lesions in the spleen, bones and gastrointestinal tract. FDG-PET/CT is also used to guide tissue sampling by identifying the most FDG avid lesions and avoiding necrotic or uninvolved tissues [13,14,40].

## 7. Germ Cell Tumors

**Figure 13:**
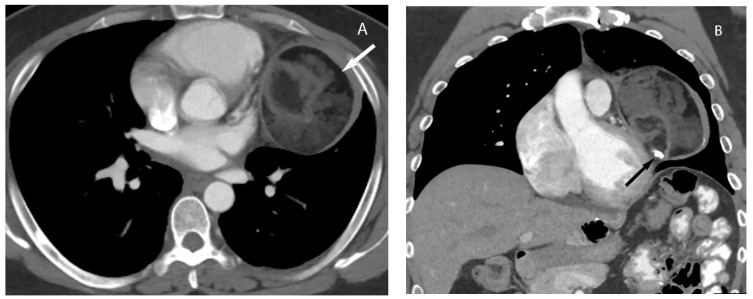
Mature teratoma. CT axial (**A**) and coronal reformat (**B**) show a well-defined mass containing soft tissue, fat (arrow in (**A**)) and calcification (arrow in (**B**)) in the prevascular mediastinum compatible with mature teratoma.

**Figure 14:**
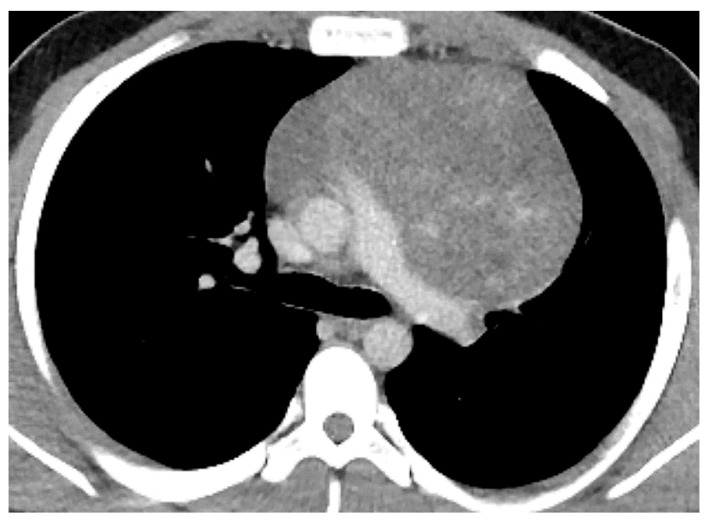
Nonseminomatous germ cell tumor (NSGCT). CT shows a large heterogeneous mass in the prevascular mediastinum. Biopsy showed teratoma with malignant transformation.

Germ cell tumors arise from primitive germ cells that fail to migrate along the urogenital ridge to the gonad and stop in the mediastinum during embryogenesis. The prevascular mediastinum is the most common extragonadal site for these tumors and comprises up to 15% of all mediastinal tumors. Histologically, these tumors are divided into teratomas, seminomas and nonseminomatous germ cell tumors (NSGCT) [28,41,42,43]. Teratomas are the most common mediastinal germ cell tumors and are composed of tissues derived from more than one of the three primitive germ cell layers. Ectodermal (teeth, skin, and hair), mesodermal (cartilage and bone) and endodermal derivatives (bronchial, intestinal and pancreatic tissue) may be present within these tumors. These tumors can be further subdivided into mature and immature teratomas, the former accounting for the majority of the teratomas. Mature teratomas are histologically well differentiated and benign while immature teratomas are considered malignant with potential for local recurrence and metastasis. Rarely, teratomas may contain intrinsic foci of carcinoma or sarcoma, in which case they are referred to as malignant teratomas. Teratomas typically affect children and young adults without gender predilection [42,44,45]. Upon CT, teratomas usually present as a well-defined heterogeneous mass with variable amounts of fat and soft tissue and may contain calcification (Figure 13). Rarely, bone or tooth can be identified within the mass. They may be predominantly cystic (unilocular or multilocular) and the presence of a fat fluid level within the mass is highly suggestive of mature teratoma but less commonly seen [4,18,46]. Other uncommon fat-containing tumors within prevascular mediastinum include thymolipoma, lipoma and liposarcoma [2,3,46]. Thymolipoma is a benign encapsulated tumor containing mostly fat and a small amount of soft tissue and fibrous septa. These tumors are present within the anatomic location of the thymus and can be very large at presentation. Lipoma is an encapsulated tumor composed predominantly of fat and contains small blood vessels. Liposarcomas also comprise predominantly fatty tissues but contain varying amounts of soft tissue and may show some aggressive features such as local invasion, locoregional lymphadenopathy and distant metastasis [25,46,47]. Seminomas and nonseminomatous germ cell tumors (NSGCT), comprising embryonal carcinoma, choriocarcinoma, yolk sac tumor, mixed germ cell tumors and teratoma with malignant transformation, are malignant germ cell tumors that occur almost exclusively in young men between the ages of 20 to 40 years. Most of the patients are symptomatic due to the large size of the tumor with compression or invasion of adjacent structures. Upon CT, these tumors usually manifest as large homogeneous or heterogeneous soft tissue masses in the prevascular mediastinum (Figure 14). Invasion of adjacent structures, metastatic lymphadenopathy and distant metastases may occur. It can be difficult to distinguish these tumors from lymphoma upon imaging and biopsy may be required to confirm the diagnosis. However, the addition of demographic and serologic information can help to narrow the differential upon imaging. Seminomas are more likely to be homogeneous upon imaging and approximately 10% of patients may show elevated serum levels of beta human chorionic gonadotropins (β-HCG) level but alpha fetoprotein (α-FP) is usually normal. NSGCTs are mostly heterogeneous upon imaging and approximately 90% of patients may present with elevated serum levels of α-FP or β-HCG [41,43,44].

### Visceral (Middle) Compartment

The visceral mediastinal compartment contains both vascular and nonvascular structures, and a variety of abnormalities may originate from these structures. The most common visceral compartment masses are lymphadenopathy, foregut duplication cysts, vascular lesions and esophageal tumors. Lymphadenopathy could be neoplastic (metastasis and lymphoma) or nonneoplastic, including infectious (tuberculosis, histoplasmosis, etc.) or inflammatory (sarcoidosis, pneumoconiosis, etc.) [2,3,48,49].

## 8. Cystic Lesions

**Figure 15:**
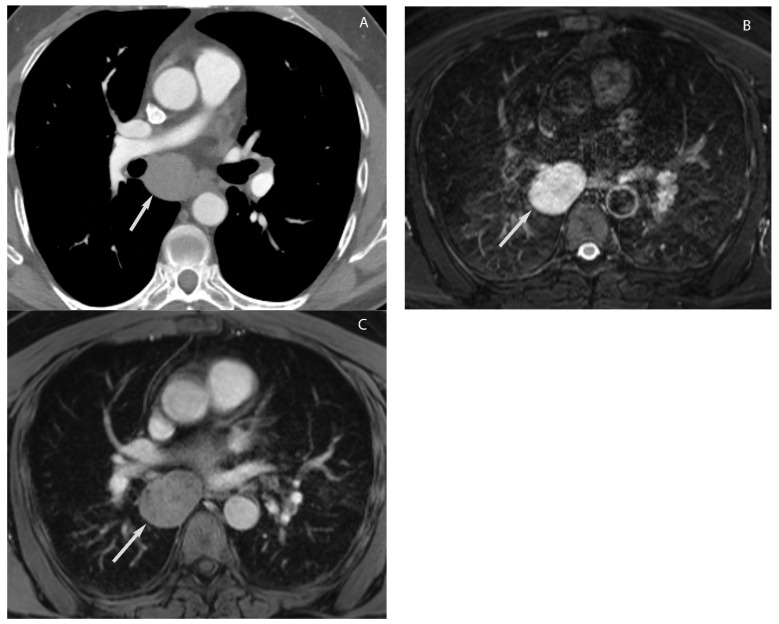
Foregut duplication cyst. CT (**A**) shows a well-marginated structure in the visceral mediastinum with attenuation values slightly higher than simple fluid. MRI (**B**,**C**) show high signal intensity upon T2W imaging (arrow in (**B**)) and no enhancement upon post-gadolinium imaging (arrow in (**C**)) consistent with fluid.

The most common cystic lesions in the visceral compartment include bronchogenic and esophageal duplication cysts. Bronchogenic cyst commonly occurs near the carina or less frequently in the right paratracheal region. Upon CT, it typically manifests as well-circumscribed, homogeneous lesions with fluid attenuation (0 to 20 HU). The cyst wall may show enhancement with contrast administration or contain calcification. Internal heterogeneity can occur in some cases due to hemorrhagic or proteinaceous contents or infection of the bronchogenic cyst. MRI is helpful in such cases to confirm the cystic nature of these legions. MRI shows high signal intensity on T2-weighted images and variable signal intensity on T1-weighted images depending upon the hemorrhagic or proteinaceous contents of the cystic lesion. Typically, these lesions do not enhance but sometimes enhancement of the wall may be seen after gadolinium administration (Figure 15) [10,11,50]. Esophageal duplication cysts also manifest as a well-circumscribed homogeneous fluid attenuation lesion adjacent to the esophagus upon CT. Internal heterogeneity may be present due to hemorrhage or infection [20,50].

## 9. Hypervascular Lesions

**Figure 16:**
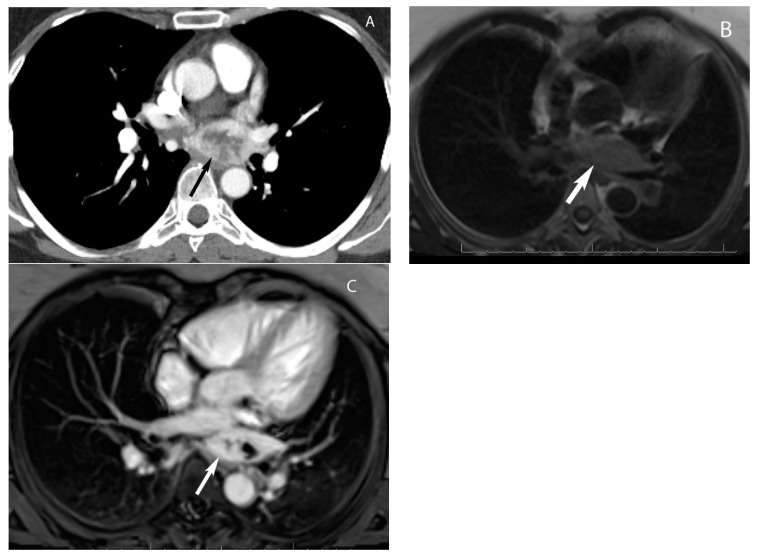
Paraganglioma. CT (**A**) shows a hypervascular mass with central necrosis in the visceral mediastinum behind the left atrium. MRI (**B**,**C**) shows the mass is hyperintense compared to muscle upon black blood double inversion MRI (arrow in (**B**)) and hypervascular upon post-gadolinium imaging (arrow in (**C**)).

Paragangliomas are rare neuroendocrine tumors that arise from chromaffin cells located outside of the adrenal gland. They can occur anywhere along the sympathetic chain or parasympathetic ganglia. These are highly vascular neoplasms that rarely occur in the visceral mediastinum, most commonly affecting the left atrium. These lesions typically manifest as homogeneously and intensely enhancing masses upon CT. Sometimes, internal heterogeneity may be present due to tumor necrosis. Upon MRI, these tumors show high signal density in T2-weighted images and may have intermediate signal intensity in T1-weighted images (Figure 16) [46,48,51,52].

**Figure 17:**
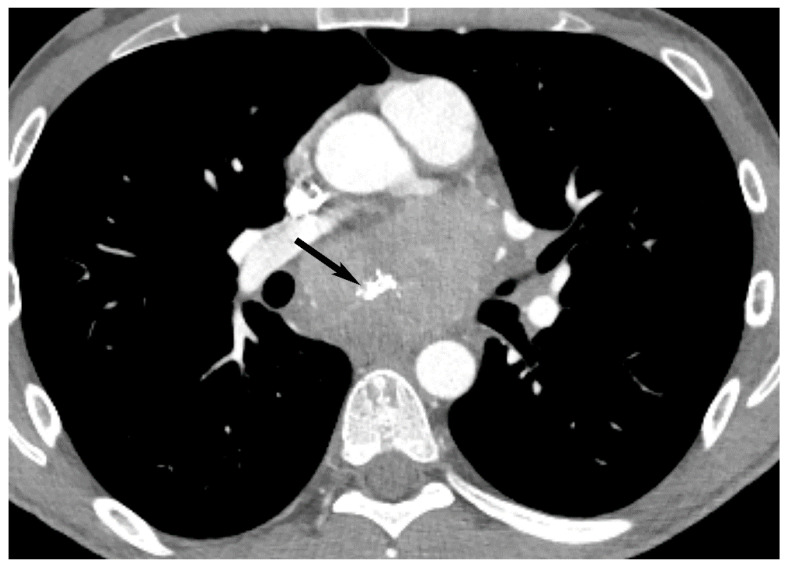
Castleman disease. CT shows hypervascular mass with a focus of calcification (arrow) in the visceral mediastinum. Biopsy showed Castleman disease.

Castleman disease, also known as angiofollicular lymphoid hyperplasia is a rare disorder characterized by nonclonal lymph node hyperplasia and frequently presents as a mediastinal mass. Castleman disease is historically divided into two forms, unicentric or multicentric, based on stations of lymph node involvement. However, the more recent histopathologic classification recognizes four different categories: hyaline vascular Castleman disease, plasma cell Castleman disease, human herpes virus 8-associated Castleman disease and Castleman disease not otherwise specified. The hyaline vascular variant is the most common, comprising 90% of cases, and mostly manifests as unicentric disease in young adults. The plasma cell variant most commonly presents as multicentric disease and tends to involve older adults. Human herpes virus 8-associated Castleman disease also commonly manifests as multicentric disease. This typically affects immunocompromised patients and has a poor prognosis [53,54,55]. Upon CT, hyaline vascular variant usually manifests as a solitary hyperenhancing mass. Rarely, calcification may be seen upon CT with punctate, coarse, peripheral or arborizing patterns (Figure 17). Upon MRI, these lesions show intermediate to slightly high signal intensity in T1-weighted images, high signal intensity in T2-weighted images and intense enhancement with gadolinium. The differential diagnosis of unicentric disease includes paraganglioma or sarcoma. Multistation lymph node enlargement is seen with multicentric disease, and enhancement with contrast is reportedly less than that for the hyaline variant. The differential diagnoses for multicentric Castleman disease include metastatic lymphadenopathy, lymphoma and sarcoidosis [53,56,57]. Histological confirmation of diagnosis is required.

## 10. Esophageal Lesions

**Figure 18:**
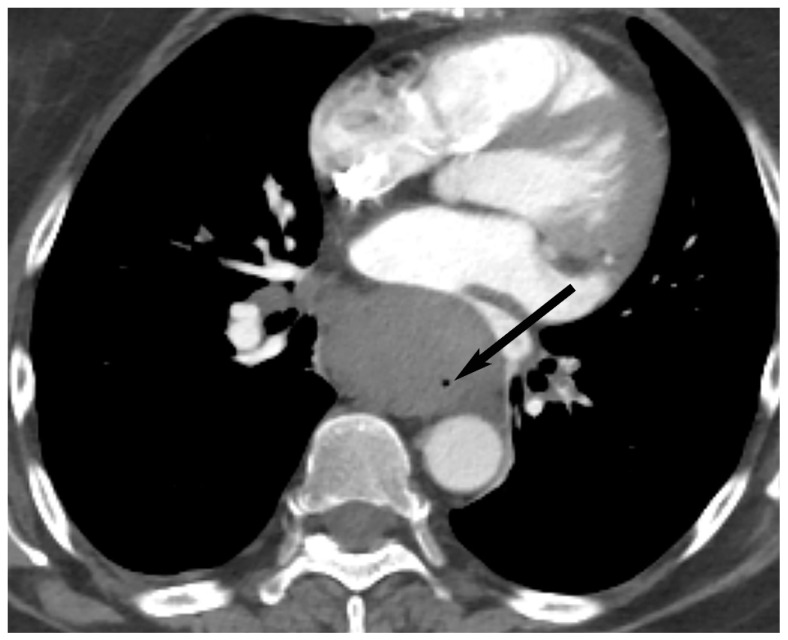
Esophageal carcinoma. CT shows a soft tissue mass in the visceral mediastinum inseparable from the esophagus (arrow). Biopsy showed esophageal carcinoma.

**Figure 19:**
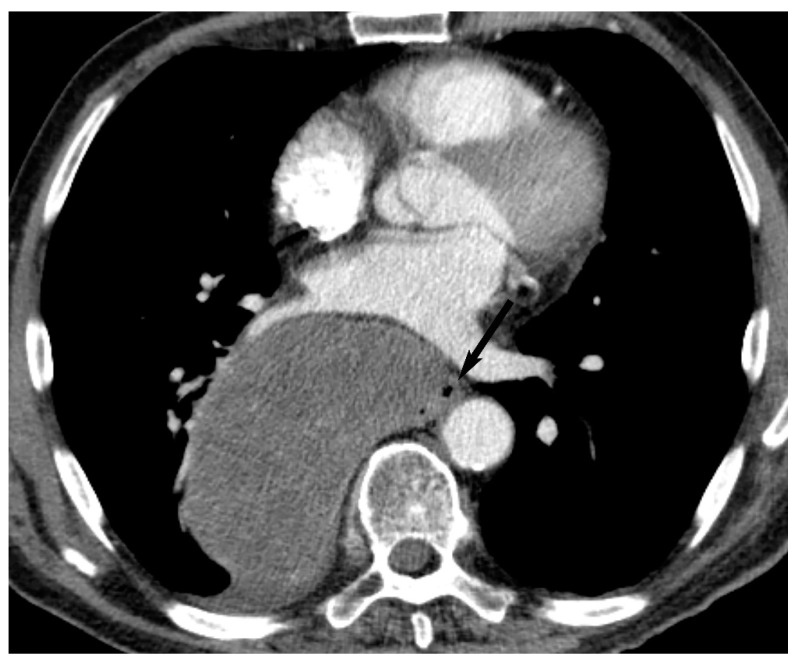
Esophageal gastrointestinal stromal tumor (GIST). CT shows a large heterogeneous mass in the visceral mediastinum inseparable from the esophagus (arrow). Biopsy showed gastrointestinal stromal tumor.

Both esophageal carcinoma and esophageal gastrointestinal stromal tumor (GIST) can present as esophageal masses and are challenging to differentiate upon imaging. Esophageal GISTs are relatively rare and more commonly found in the stomach or small intestine. Esophageal carcinomas often present as irregular or infiltrative masses that can invade the adjacent structures (Figure 18). GISTs, on the other hand, tend to have a well-defined or lobulated shape and smooth margins. GISTs are mesenchymal tumors that usually arise in the submucosal layer of the esophageal wall. Therefore, they may show an extraluminal growth pattern that can cause luminal narrowing or displacement (Figure 19). In contrast, esophageal carcinomas arise from the mucosal layer and tend to infiltrate the wall, resulting in concentric growth and wall thickening. Upon contrast-enhanced images, esophageal carcinomas typically show heterogeneous enhancement whereas GISTs may show a homogeneous enhancement pattern. It is important to note that histopathological evaluation is necessary for accurate diagnosis [58,59].

### Paravertebral (Posterior) Compartment

**Figure 20:**
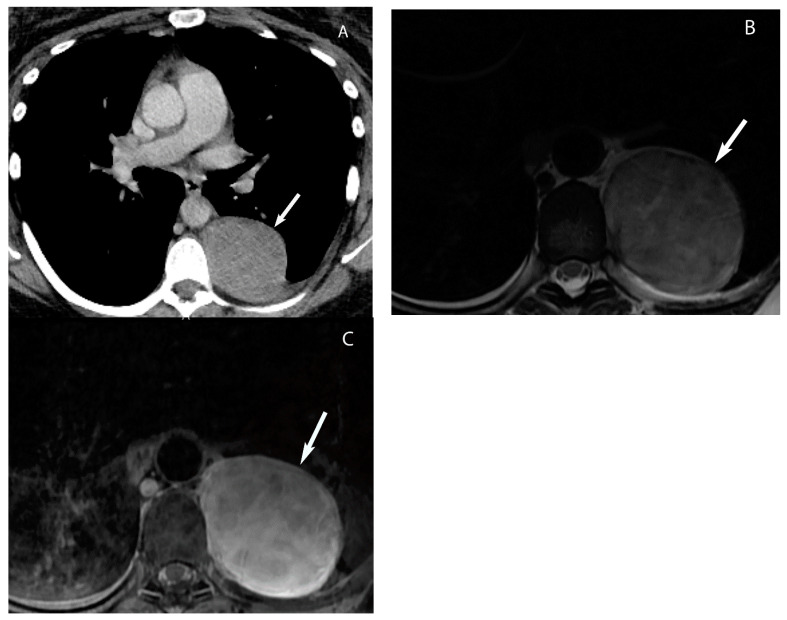
Schwannoma. CT (**A**) shows a well-defined spherical shaped mass in the left paraspinal region (arrow). MRI (**B**,**C**) shows the mass is hyperintense upon T2W imaging (arrow in (**B**)) and enhances with gadolinium (arrow in (**C**)).

Most of the paravertebral masses are neurogenic in origin. Peripheral nerve sheath tumors including schwannomas and neurofibromas are common in adults. Tumors of the autonomic nervous system such as ganglioneuromas, ganglioneuroblastomas and neuroblastomas are common in children and young adults. In addition to patient age, the shape of the tumors can be of helpful in differentiating peripheral nerve sheath tumors from sympathetic ganglion cell tumors. Peripheral nerve sheath tumors are spherical or dumbbell in shape whereas sympathetic ganglion cell tumors are vertically elongated or cylindrical in shape. MRI is the imaging modality of choice for characterizing and assessing the intraspinal extension of these tumors [49,60,61] (Figure 20).

**Figure 21:**
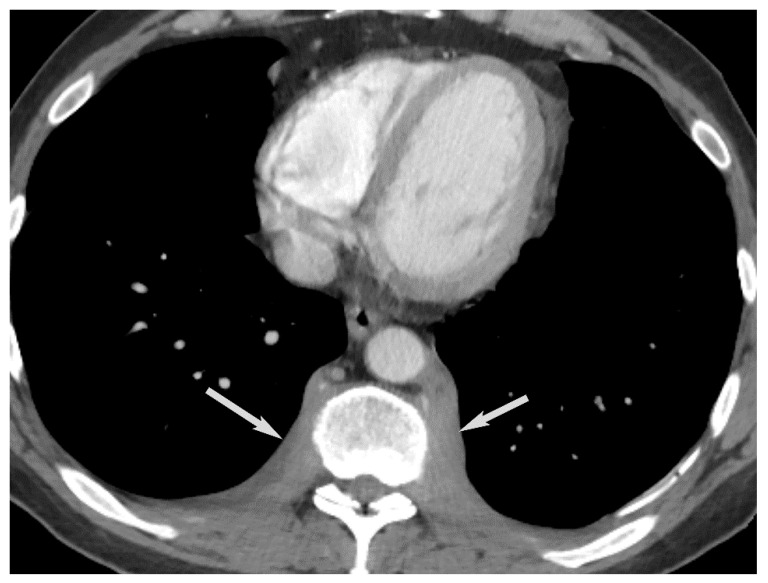
Extramedullary hematopoiesis. CT shows bilateral paraspinal soft tissue thickening (arrows) in a patient with leukemia and anemia.

Primary or metastatic osseous tumors and nonneoplastic lesions such as extramedullary hematopoiesis are other less common pathologies that can be seen in the paravertebral compartment. Extramedullary hematopoiesis should be suspected when a paraspinal soft tissue mass is seen in patients with underlying hematologic conditions and anemia. These can be unilateral or bilateral and show intense enhancement with contrast similar to the spleen (Figure 21). In long-standing cases when a patient’s hematocrit returns to normal, the yellow marrow takes over, and fatty attenuation may be seen within the masses [49,60,62].

## 11. Conclusions

A wide variety of benign and malignant conditions can occur in the mediastinum. Localization to the mediastinal compartment, patient demographics and imaging findings are extremely helpful in creating meaningful differential diagnoses.

## Figures and Tables

**Table 1:** ITMIG classification of mediastinal compartments.

Compartment	Boundaries
Prevascular	Anterior: sternum
	Posterior: anterior surface of pericardium as it wraps around the heart
	Superior: thoracic inlet
	Inferior: diaphragm
	Lateral: mediastinal pleura
Visceral	Anterior: posterior boundaries of prevascular compartment
	Posterior: vertical line connecting a point on each thoracic vertebral body 1 cm posterior to its anterior margin
	Superior: thoracic inlet
	Inferior: diaphragm
Paravertebral	Anterior: posterior boundaries of the visceral compartment
	Posterolateral: vertical line against the posterior margin of the chest wall at the lateral margin of the transverse process of the thoracic spine
	Superior: thoracic inlet
	Inferior: diaphragm
